# New perspective for the upscaling of plant functional response to flooding stress in salt marshes using remote sensing

**DOI:** 10.1038/s41598-024-56165-4

**Published:** 2024-03-05

**Authors:** Marco Vuerich, Paolo Cingano, Giacomo Trotta, Elisa Petrussa, Enrico Braidot, Dora Scarpin, Annelore Bezzi, Michele Mestroni, Elisa Pellegrini, Francesco Boscutti

**Affiliations:** 1 DI4A Department of Agricultural, Food, Environmental and Animal Sciences, University of Udine, 33100 Udine, Italy; 2 NBFC, National Biodiversity Future Center, 90133 Palermo, Italy; 3 Department of Environmental and Life Sciences (DSV), University of Trieste, 34127 Trieste, Italy; 4 Department of Mathematics and Geosciences, University of Trieste, 34128 Trieste, Italy; 5 Agricoltura Innovativa Mestroni, 33036 Mereto di Tomba, UD Italy

**Keywords:** Community ecology, Plant ecology, Plant stress responses, Wetlands ecology

## Abstract

Understanding the response of salt marshes to flooding is crucial to foresee the fate of these fragile ecosystems, requiring an upscaling approach. In this study we related plant species and community response to multispectral indices aiming at parsing the power of remote sensing to detect the environmental stress due to flooding in lagoon salt marshes. We studied the response of *Salicornia fruticosa* (L.) L. and associated plant community along a flooding and soil texture gradient in nine lagoon salt marshes in northern Italy. We considered community (i.e., species richness, dry biomass, plant height, dry matter content) and individual traits (i.e., annual growth, pigments, and secondary metabolites) to analyze the effect of flooding depth and its interplay with soil properties. We also carried out a drone multispectral survey, to obtain remote sensing-derived vegetation indices for the upscaling of plant responses to flooding. Plant diversity, biomass and growth all declined as inundation depth increased. The increase of soil clay content exacerbated flooding stress shaping *S. fruticosa* growth and physiological responses. Multispectral indices were negatively related with flooding depth. We found key species traits rather than other community traits to better explain the variance of multispectral indices. In particular stem length and pigment content (i.e., betacyanin, carotenoids) were more effective than other community traits to predict the spectral indices in an upscaling perspective of salt marsh response to flooding. We proved multispectral indices to potentially capture plant growth and plant eco-physiological responses to flooding at the large scale. These results represent a first fundamental step to establish long term spatial monitoring of marsh acclimation to sea level rise with remote sensing. We further stressed the importance to focus on key species traits as mediators of the entire ecosystem changes, in an ecological upscaling perspective.

## Introduction

Coastal areas are considered among the most valuable but endangered ecosystems globally due to their susceptibility to global change and other anthropogenic factors^1–3^. Among coastal environments, salt marshes represent the most important sea-land transitional ecosystem of estuarine and lagoon systems^4–7^, undergoing rapid alterations due to climate change aftermaths^8,9^. Climate-induced sea level rise (SLR) and storm surges threaten the integrity and functionality of salt marshes by increasing the frequency and intensity of flooding stress^3,10,11^. For SLR in particular, the salt marsh survival depends on their accretion ability in relation to the sediment inputs and flooding stress increase^12,13^. In fact, any reduction in plant growth may affect the capacity of the marsh to attenuate waves (especially in storms) and trap sediment^14^, influencing salt marsh accretion^15^. Moreover, decreased root or rhizome production may weaken marsh bank stability leading to increased susceptibility to erosion^16,17^. The ultimate consequence is the salt marsh area shrinkage and, eventually, its migration or disappearance^18^. For these reasons understanding how salt marsh plants acclimate to flooding and monitoring such processes is of the utmost importance in depicting future scenarios and possible mitigation actions.

Tidal flooding is a pivotal factor driving plant species growth, distribution, and zonation in salt marshes^19–22^. In an equilibrium state, tidal flooding is mainly determined by land morphology^23^ and soil texture (i.e., soil permeability)^24^. As a consequence, flooding influences crucial soil features, such as the availability of oxygen^25,26^, triggering intense plant-soil feedbacks^27^. An increase of flooding stress might cause the reduction of plant performance in a soil-mediated process that can have repercussions on the overall biodiversity and productivity^27^.

Plant-soil feedbacks are often modulated by plant functional traits of key species of the community^27,28^, that in salt marsh plants are represented by both morphological and physiological traits determining the phenotypic response of a plant to flooding^29–31^. A functional traits approach is hence crucial to predict the response of key species of the ecosystems, which changes could also affect the entire ecosystem processes and properties (e.g., nutrient cycles, biodiversity)^32,33^, opening important upscaling perspectives.

Among the most promising tools for scale-up of ecological processes, remote sensing has been used across different ecological scales and systems^34–38^. In salt marsh, application of remote sensing tools (e.g., satellite and unmanned aerial vehicle UAV images) has been limited to plant community discrimination^39,40^, plant phenology detection^41,42^, plant-plant interactions^43^ and to survey overall ecosystem properties^44^. In these ecosystems, while remote sensing has been applied for the monitoring of some gross ecosystem properties (e.g., net primary production), linking the mechanistic response of individual plants (e.g., growth traits, physiological response) to the ecosystem level remains unexplored and promising.

We studied the response of salt marsh plant communities by upscaling the functional response of the key species *Salicornia fruticosa* (L.) L. across a flooding gradient in a lagoon system. We further linked the functional response of *S. fruticosa* (i.e., secondary metabolites, plant growth traits) to multispectral indices with the aim to extend the potential effect of plant response to a broader spatial and ecological scale.

We expected that high flooding stress has a direct effect on *S. fruticosa* response and the properties of the whole salt marsh ecosystem. We did expect flooding to shape the phenotypic plasticity of *S. fruticosa* by reducing individual growth traits (i.e., stem elongation), photosynthetic pigment contents, and increasing secondary metabolites production (flavonoids, betacyanins). We expected a decrease of community productivity (biomass) and biodiversity with an increase of the stress (flooding). We also explored the potential of high-resolution multispectral image acquisition to depict the link between traits and community response to the environmental stress. We finally expected multispectral indices to be affected by both community and key species functional traits, in relation to an alteration of vegetation spectral firm induced by individual mean functional changes.

## Results

### Salt marsh plant community response to flooding and soil texture gradient

**Figure 1:**
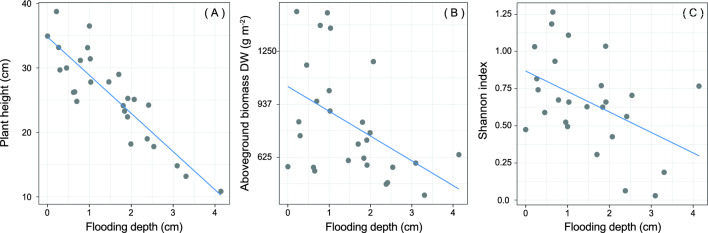
Effect plots of flooding depth on salt marsh plant community traits, namely plant height (**A**), aboveground biomass dry weight (DW), (**B**) and Shannon index (**C**).

All the community traits mean and range values are shown in Table 1. Community plant height, dry weight and plant diversity (i.e., Shannon index) decreased along the flooding gradient while they were not significantly affected by soil clay content (Table 2, Fig. 1a–c) (respectively 18.0%, 27.4%, 13.4% of the total variance explained). At higher levels of flooding, plant communities were short (i.e., mean plant height) and with a low overall aboveground biomass (i.e., total dry weight). In contrast, plant community dry matter content was not affected neither by flooding depth nor by soil clay content (Table 2; R^2^ = 4.8%).

**Table 1:** Descriptive statistics of environmental, plant community and *Salicornia fruticosa* variables.

Variable	Mean	Std. dev	Max	Min
Flooding depth (cm)	1.59	1.03	4.14	0.01
Clay content (%)	19.55	6.26	30.91	5.84
Species richness	4.15	1.38	8	2
Shannon index	0.69	0.35	1.47	0.06
Salicornia fruticosa cover value (%)	74.45	16.75	99.01	28.37
Plant height (cm)	25.87	6.89	42.00	14.80
Total dry weight (g)	131.07	54.83	264.00	64.2
Total dry matter content	320.48	58.35	429.52	214.61
Shoot dry weight (g)	0.03	0.01	0.05	0.02
Shoot dry matter content	139.68	16.67	174.93	110.11
Shoot length (cm)	4.10	0.89	6.76	2.64
Chlorophyll concentration (μg g^−1^)	481.66	139.27	877.63	286.39
Pheophytin content	32.19	14.42	68.18	16.28
Carotenoid concentration (μg g^−1^)	90.58	12.03	113.60	64.02
Flavonoid concentration (μg g^−1^)	23.86	8.40	40.83	7.96
Betacyanin concentration (μg g^−1^)	24.30	15.18	60.57	2.91
NDVI	0.47	0.08	0.64	0.34
LCI	0.16	0.04	028	0.09
RGRI	0.95	0.08	1.10	0.78
ARI	8.64	1.38	11.25	6.31

**Table 2:** Results of the LMMs relating the salt marsh community traits (i.e., plant height, total dry weight, total dry matter content, Shannon index) with flooding depth and soil clay content.

Dependent variable	Independent variable	Df	Estimate	SE	t-value	p-value
Plant height	Flooding	1,16	− 5.93	1.20	− 4.92	** < 0.001**
	Clay content	1,16	0.07	0.17	0.40	0.698
Total dry weight	Flooding	1,16	-23.33	9.21	− 2.53	**0.022**
	Clay content	1,16	2.09	1.53	1.36	0.191
Total dry matter content	Flooding	1,16	− 1.97	11.19	− 0.18	0.863
	Clay content	1,16	1.97	1.86	1.06	0.306
Shannon index	Flooding	1,15	− 0.14	0.06	− 2.48	**0.026**
	Clay content	1,15	− 0.01	0.01	− 1.09	0.295

Significant relationships are in bold (p < 0.05). Degrees of freedom (Df), estimate, standard error (SE), t-value and p-values are shown.

### *Salicornia fruticosa* response to flooding and soil texture gradient

**Figure 2:**
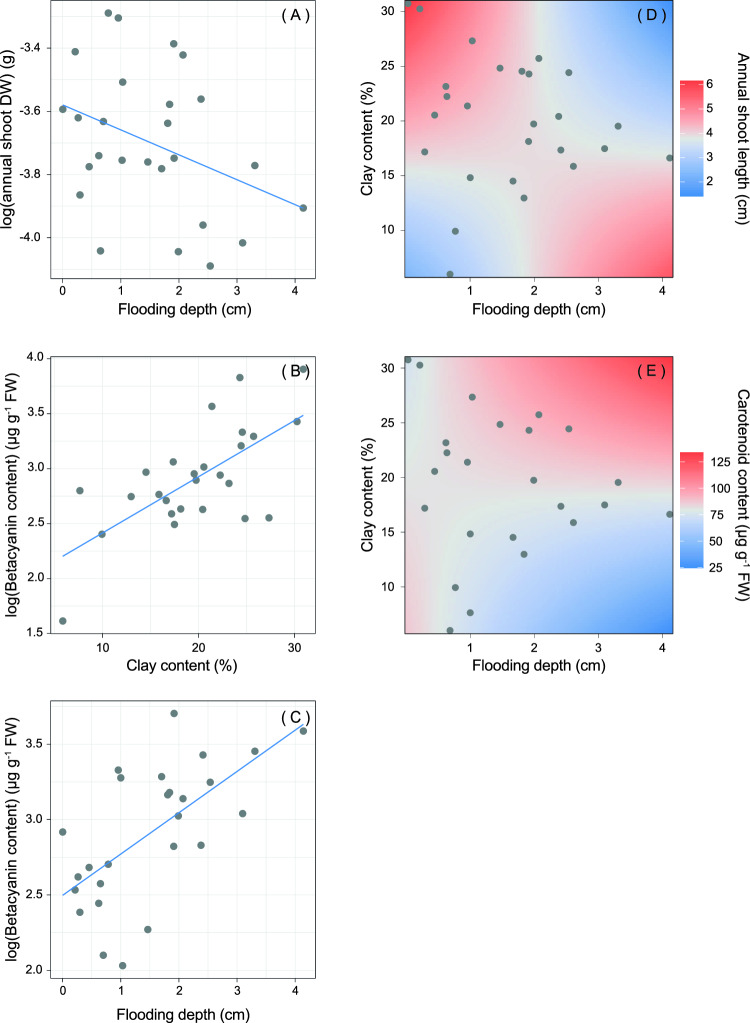
Effect plots of flooding depth, clay content and their interaction on *S. fruticosa* traits. Changes of annual shoot dry weight (DW) (**A**) betacyanin content (**B**, **C**) annual shoot length (**D**) and carotenoid content (**E**) along the flooding gradient, the clay content, and their interaction.

All the *S. fruticosa* trait mean and range values are shown in Table 1. The functional response of *S. fruticosa* was mainly modulated by flooding depth and its interaction with soil texture (i.e., clay content) (Table 3). *S. fruticosa* shoot dry weight showed a significant decrease along the flooding depth gradient (Fig. 2a, R^2^ = 18.4%), whereas betacyanin shoot content was also significantly affected by soil clay content, showing a significant positive relationship (Table 3, Fig. 2b,c; R^2^ = 25.5%). *S. fruticosa* shoot length and carotenoid content were shaped by the interaction between flooding depth and soil clay content (Table 3; R^2^ = 58.4% and 28.2%). In particular, in soil with high content of clay the shoot length sharply decreased along the increase of flooding depth, while in soils poor in clay (i.e., sandy soils) the increase of flooding depth slightly increased the annual plant stem elongation (shoot length) (Fig. 2d). A high stem carotenoid content was caused by high flooding depth levels and high percentage of clay in the soil, whereas a low carotenoid content was observed in more sandy soil more subjected to flooding (Fig. 2e). As for total dry matter content of plant community, also shoot dry matter content was affected neither by flooding nor by clay soil content (Table 3; R^2^ = 8.3%). Chlorophyll stem content was also not affected by the abiotic gradients (R^2^ = 26.8%).

**Table 3:** Results of the LMMs relating the *S. fruticosa* traits (i.e., shoot dry weight, shoot dry matter content, shoot length, chlorophyll, carotenoids, flavonoids, betacyanin content) with flooding depth, soil clay content and their interaction.

Dependent variable	Independent variable	Df	Estimate	SE	t-value	p-value
Log (Shoot dry weight)	Flooding	1,16	− 0.11	0.06	− 2.05	**0.050**
	Clay content	1,16	0.01	0.01	1.16	0.265
Shoot dry matter content	Flooding	1,16	− 1.05	3.41	− 0.31	0.762
	Clay content	1,16	0.11	0.51	0.20	0.841
Shoot length	Flooding	1,14	1.18	0.54	2.20	**0.046**
	Clay content	1,14	0.15	0.03	4.69	** < 0.001**
	Flooding × clay content	1,14	− 0.08	0.03	− 2.77	**0.015**
Chlorophyll	Flooding	1,16	− 6.39	25.60	− 0.25	0.806
	Clay content	1,16	− 4.09	3.74	− 1.09	0.290
Carotenoids	Flooding	1,12	− 31.19	8.93	− 3.49	**0.004**
	Clay content	1,12	− 0.85	0.44	− 1.94	0.076
	Flooding × clay content	1,12	1.56	0.48	3.29	**0.007**
Flavonoids	Flooding	1,16	− 0.29	1.60	− 0.18	0.858
	Clay content	1,16	− 0.34	0.27	− 1.26	0.226
Log (Betacyanin)	Flooding	1,15	0.27	0.13	2.27	**0.038**
	Clay content	1,15	0.05	0.02	2.97	**0.010**

Significant relationships are in bold (p < 0.05). Degrees of freedom (Df), estimate, standard error (SE), t-value and p-values are shown.

### Upscaling of plant community and *S. fruticosa* functional responses by means of remote sensing signals

Flooding depth significantly reduced NDVI while RGRI was positively related with this environmental variable (Table S2). ARI and LCI were shaped by the interaction between flooding depth and soil clay content, showing lowest values in conditions of high flooding depth and clayey soil.

**Figure 3:**
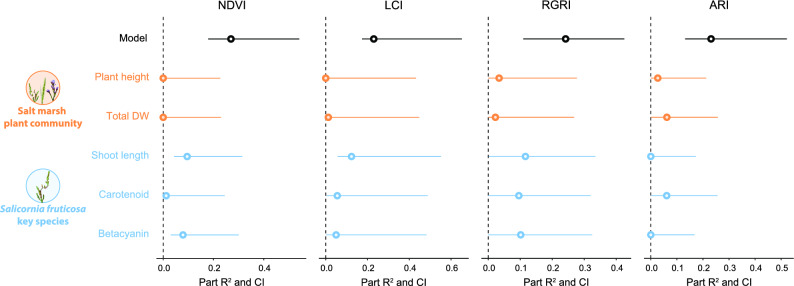
Contribution of salt marsh plant community traits and *S. fruticosa* traits to vegetation indices. Proportions of vegetation indices (i.e., NDVI, LCI, RGRI, ARI) variance explained by plant community traits (i.e., plant height and total dry weight) and *S. fruticosa* traits (i.e., shoot length, carotenoid and betacyanin content).

For NDVI about 27% of the total variance was explained by plant traits (confidence interval, hereafter CI [0.17–0.60]) while community trait (i.e., total dry weight and height) did not contribute (Fig. 3, Table S3). Most of the variance was assigned to shoot length (part R^2^ = 0.10, CI [0.00–0.23]) and betacyanin shoot content (part R^2^ = 0.08, CI [0.00–0.22]).

Plant traits explained 23% of the LCI total variance (CI [0.13–0.44]) (Fig. 3, Table S3). Shoot length was the main trait contributing the variance (part R^2^ = 0.12, CI [0.00–0.23]) along with carotenoids (part R^2^ = 0.06, CI [0.04–0.28]) and betacyanin shoot content (part R^2^ = 0.05, CI [0.03–0.28]).

The 37% of the variance of RGRI mainly explained by shoot length, carotenoid and betacyanin content (namely part R^2^ = 0.12, CI [0.05–0.34]; part R^2^ = 0.10, CI [0.04–0.32]; part R^2^ = 0.10, CI [0.04–0.33]), (Fig. 3, Table S3).

For ARI about 23% of the total variance was explained only by community and *S. fruticosa* traits (CI [0.16–0.39]). Community dry weight and carotenoid shoot content were the variables mostly contributing to this proportion (both part R^2^ = 0.06, CI [0.04–0.19]). Community plant height explained only 3% of the variance (CI [0.01–0.15]), whereas annual shoot length and betacyanin content did not affect this index (Fig. 3, Table S3).

## Discussion

Our findings confirmed flooding depth to be the main driver shaping plant communities in tidal salt marshes. Interestingly, flooding depth was the only driver directly affecting plant community features (i.e., biomass, plant height, and diversity), while its interaction with soil clay content shaped the functional response of *Salicornia fruticosa* (i.e., shoot length, shoot carotenoid content), probably via increasing soil anoxia soil^24^.

By exploring the relationships between plant community and *S. fruticosa* functional traits and high-resolution remote sensing derived indices we found key species traits, in particular stem length and pigment content (i.e., betacyanin, carotenoids), to be more effective than others for an upscaling perspective in tidal salt marshes. This suggests that the response of the key species, already demonstrated to be important drivers for the modulation of the whole ecosystem responses to environmental changes^27,32^, should not be neglected in the upscaling process of the vegetation response to flooding, from the plot to ecosystem level.

### Saltmarsh response to flooding and soil texture gradient

#### Community response

An increase of flooding depth produced a significant decrease in plant diversity, biomass and plant height. Flooding depth has been already linked to the reduction of salt marsh productivity, by modulating plant growth^45,46^. Instead, plant community DMC was not affected by flooding depth, showing a decoupling between this index and the community dry biomass. This suggests that saltmarsh plants under abiotic stress reduced the growth containing the changes in the overall rate between tissues and water content (i.e., DMC), as probable need to cope with reduce evapotranspiration and osmotic balance^47,48^.

Clay content did not affect plant community traits, while it contributes for the shaping of *S. fruticosa* response, that, in turn, can facilitate other species along the abiotic stress gradient caused by flooding depth and soil clay, causing plausible feedback at the community level^24,49^.

#### *Salicornia fruticosa* response

As hypothesized flooding depth significantly affected *S. fruticosa* growth and physiological response, but its effect was often modulated by soil texture (i.e., clay content).

Deeply flooded plants of *S. fruticosa* showed a low increment in annual biomass (i.e., annual shoot dry weight), while their elongation (shoot length) was finely modulated by the interaction of clay content and flooding depth, showing the lowest values in sites deeper flooded and with high soil clay content. These findings suggest that elongation of plants, often used to avoid complete submergence, is used by plants only in sites where soil conditions are less harsh (i.e., sandy soils where complete anoxia is rarely reached), while plants could be severely limited in growth in compact clayey soils, including their shoot elongation^50^. This is induced by the variation of parameters such as (i) the limited diffusion of O_2_ and CO_2_^51^, (ii) the increase of toxic volatile substances^52^, (iii) the limitation of nutrients^53^, and (iv) the increase in osmotic stress. In this light, the elongation of *S. fruticosa* annual shoot could represent a common escape strategy to cope with submergence, shared with other wetland plants^27,54^.

As expected, deep flooding stimulated the production of secondary pigments such carotenoids but did not affect chlorophyll content of photosynthetic stems. Other contributions showed carotenoids and chlorophyll content to be particularly sensitive to flooding depth^55,56^. Here we found that carotenoids reached their maximum values when deep flooding was combined with high clay content in the soil, consistently with what found in other species^57^, as probable response to hypoxia^58,59^. Instead on highly permeable soils the flooding induced a decrease of carotenoids, this was consistent to what evidenced in other studies where the mere application of flooding decreased all pigments production, carotenoids included^55,56^. It is thus probable that only a strong soil hypoxia due to the interplay of deep flooding and soil clay content can activate the protective role of a high carotenoid concentration. In addition, the complete submersion of aerial tissues has been observed to lead to a change in the ratio between the content of chlorophyll and carotenoid, as seen in *Sarcocornia perennis* (Mill.) A. J. Scott^60^. Flooding did not affect flavonoids, while a greater content of betacyanins was found in *S. fruticosa* plants growing on clayey soils and with deep flooding. Flavonoids seem to play in response to water-related stress^61,62^, while were demonstrated to be primarily related to soil hypoxia^63^. Albeit not significant, we found an increase of flavonoids in soils with more clay content, and hence, more subjected to lack of oxygen.

Such as for anthocyanins, betacyanins accumulation in vegetative organs has been generally related to their protection from environmental stress^60,64^. Consistently, we observed an increase of betacyanins under flooding stress in soil with high clay content that might be related to their capacity to attenuate effects of soil hypoxia on plants as effective scavengers of reactive oxygen species and osmotic photo-protectants^65–67^.

### Potential upscaling of plant response by remote sensing

Multispectral images have been already used to indirectly quantify the submersion in salt marsh vegetation^68^. Here, we evidenced the selected multispectral indices might be useful to monitor of the direct flooding effect, or mediated by the soil clay content, on the key species response. Despite the low explained variance, that may be attributable to the limited radiometric and spectral resolutions of the multispectral camera used, this result, improved in its effectiveness and accuracy, suggests the plausible use of remote sensing tools to monitor the acclimatization mechanisms of these communities. In particular, the rate of sea level rise (1.5 mm year^−1^) in the norther Adriatic Sea is consistent with our narrow tidal frame, also in relation to the saltmarsh accretion, deemed to attenuate the effects of sea rise^69^. Despite the narrow flooding gradient species and community responses were remarkable, therefore offering a wide array of plant and community responses. We found *S. fruticosa* growth (i.e., stem length) and physiological traits (i.e., betacyanin, carotenoids) to be the plant traits that mostly explained multispectral derived indices. Many authors already evidenced the direct relationship between indices and the whole community response to flooding, proposing for instance NDVI for the quantification of biomass and other ecosystem properties^70–72^. Instead, we found the individual response of the key species *S. fruticosa* to be more important than the whole plant community in determining the multispectral signal of vegetation: although the variance explained individually by them is relatively low, together they contribute to the total explanation of this vegetation index. The community response to flooding (i.e., biomass, plant diversity) might be related to the reduction in the biomass of the key species *S. fruticosa*, as suggested by previous results in similar communities^27^, suggesting a possible upscaling link between the studied species and the response of the entire ecosystem, regardless of the environmental stress intensity considered. This is indicating that remote sensing monitoring over time might reveal the acclimation of saltmarshes to sea level rise by intercepting the response of the key species *S. fruticosa*, opening new perspective in the multispectral image interpretation. Salt marsh key species has been already linked to the community response by plant trait-soil interactions^24,27^. Our findings suggest that these feedbacks might also drive the remote sensing-vegetation relationships for the monitoring of the ecosystem properties. Moreover, these relationships suggest for a strong complexity occurring between the soil-individual-community responses, also involving facilitation/competition interactions^24^. This complexity probably contributes to determining the low variance of the remote sensing indices explained by singular traits of the key species and the community. NDVI is one of the most used indices for numerous upscaling purposes^9,73^. The application of NDVI in our sites was informative about the *S. fruticosa* growth response and betacyanin stem content. We further suggest that other indices, namely LCI, RGRI and ARI might improve the upscaling of salt marsh response to flooding. The LCI was mainly explained by plant growth and at a lesser extent by carotenoids and betacyanins. This index was originally proposed as very sensitive for estimation of chlorophyll content and distribution in leaves. In our study area it seems to better represent the overall status of the *S. fruticosa*, similarly to RGRI that showed similar, but higher partial regression values for the physiological and growth traits. ARI captured most of the carotenoid signal, being lower at high concentrations of carotenoids. ARI is mainly used for detection of anthocyanin content^74^, but in our study it appears to better evidence changes in carotenoids and community biomass. This might be related either to a negative relationship between anthocyanins (not measured) and carotenoids or to a species-specific shift in plant reflectance due to its succulent growth form^75^. These results also suggest to further investigate the relationship between stress and modification of the spectral signature of plant species, that could greatly enhance the strength of the relationships between remote sensing indices and plant responses or suggest for the development of new indices more suitable for this kind of environmental stress and habitat.

In salt marsh halophytes, a decisive role could be played by the accumulation of pigments and other metabolites that can also shift a sensible visible change in plant chromaticism^30,76^. We only partially parsed such a complex physiological response, which study in the future could increase the upscaling perspectives of plant eco-physiological acclimation to abiotic stresses.

## Conclusions

We suggest remote sensing as a promising tool able to potentially detect acclimation response of halophytes community and ultimately to merge different ecological scales, proving new achievements for the understanding of spatial distribution and to forecast the vegetation changes due to abiotic stress gradient. In this light, our fine-tuning monitoring of the flooding effect on the key species response might be useful to monitor the acclimation mechanisms by remote sensing tools, being consistent with local sea level rise rates. Our findings might represent a novel approach to assess and monitoring the impacts of the ongoing rising sea level rise in wetland areas. We evidenced that the ongoing sea level rise can lead to a progressive reduction of plant cover and biomass, as well as the height of the populations, by increasing the areas subjected to high levels of submersion stress. Nonetheless, further improvements are needed to better investigate the changes in spectral signature of plant species under stress, to develop novel multispectral indices and hence enhance the power of remote sensing to spatially monitor these environmental stresses and the habitat response. We also highlighted that some physiological processes such as the accumulation of pigments and secondary metabolites in relation to increasing levels of stress could play a fundamental role for future remote sensing monitoring and upscaling. These finding suggest that the acclimation of saltmarshes to sea level rise could be revealed through remote sensing monitoring over time by intercepting the response of the key species *S. fruticosa*. These upscaling perspectives applied to 'sentinel ecosystems', such as salt marshes, might provide an early warning on global and regional changes, with fundamental understanding of future scenarios of the entire coastal system.

## Materials and methods

### Study area and sampling design

**Figure 4:**
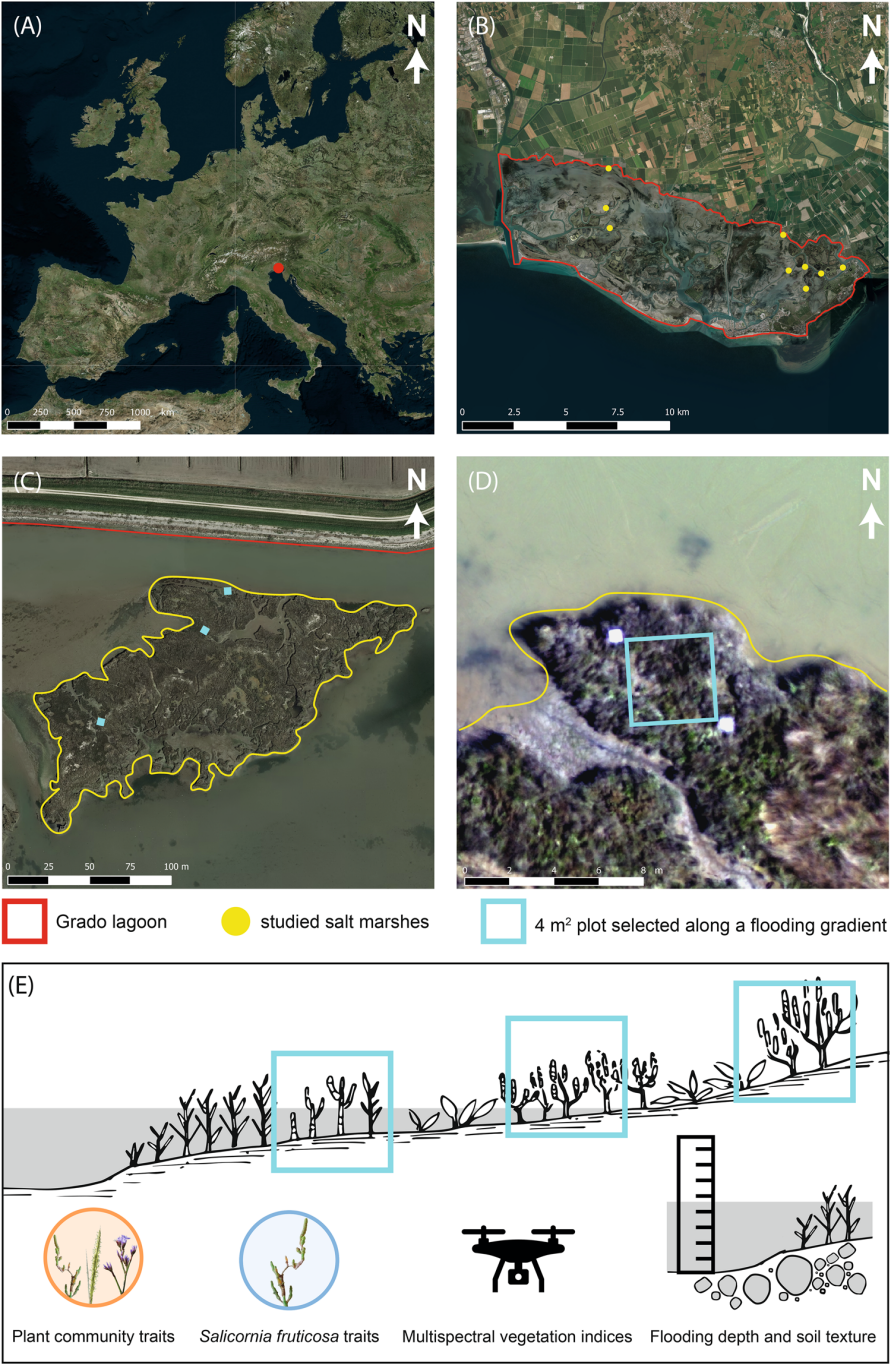
The study area of the Grado lagoon in the northern Adriatic Sea (**A**), the 9 studied salt marshes (**B**), the 3 sampling plots chosen along a flooding gradient in one of the studied salt marshes (**C**), the 4 m^2^ plot delimited by ground control point (**D**). and the experimental design (**E**).

The study was conducted in the Grado lagoon (from 45°42′10.5ʺN 13°9′17.8ʺE to 45°40′49.8ʺN 13°21′31.2ʺE) in the Northern Adriatic Sea (Friuli Venezia Giulia, Italy) (Fig. 4a). The site is designated as both a Special Area of Conservation (SAC) and a Special Protection Area (SPA) in the Natura 2000 network (site code IT3320037). The mean annual rainfall is 974 mm, with an average temperature ranging from 3.1 °C in January to 29.0 °C in July. The lagoon is morphologically classified as a leaky lagoon^77^. It is strongly influenced by tides which are semi-diurnal, with a mean range of 0.65 m and spring and neap ranges of 1.05 m and 0.22 m, respectively^78^.

The sampling was performed during May 2021 in 9 salt marshes (Fig. 4b). In each salt marsh, 3 sampling areas (plots) of 4 m^2^ (2 m × 2 m) (Fig. 4c,d) were chosen along a flooding gradient according to ground micro-morphology of salt marshes, for a total of 27 plots. The sampling design scheme is reported in Fig. 4e.

### Flooding and soil texture

The flooding depth and flooding duration were measured at each sampling site (27 plots) by using a water level data logger (HOBO U-20L-01, Onset, resolution: 0.02 kPa, 0.21 cm; water level accuracy ± 0.1%). The sensors were positioned at the ground level, with a recording interval of 5 min along a complete tidal cycle (2 weeks, including a shift from spring tide to neap tide). For an accurate flooding measurement, in each monitored saltmarsh a reference datalogger was positioned in high elevation point never subjected to water submersion. Pressure data, recorded with the loggers, were automatically compared with reference air pressure measurements, and hence, converted to the height of the water column submerging the soil, using the HOBOware®Pro software (version 3.7.14, Onset). Flooding depth was expressed as the mean height (cm) of the water column of a complete tidal cycle, while the flooding duration as the percentage of time the logger was flooded during a complete tidal cycle. We used mean depth to better represent the average conditions of water column. However, we verified the occurrence of a high correlation between mean height and maximum height of water depth (r = 0.89; p < 0.001), considering them interchangeable. The studied tidal range (Table 1) represent a significant shift in ecological conditions and was already demonstrated to trigger remarkable changes in saltmarshes plant communities^27^. Moreover, the range is also representing a good proxy of the sea level rise that in the norther Adriatic Sea have an increasing rate of 1.5 mm year^−1^ over the last decades.

A soil sample was collected at the center of each plot using a cylindrical tube (height: 12 cm, width: 3.5 cm, volume: 115.5 cm^3^), transported to the laboratory for soil texture analyses. All soil samples (n = 27) were processed by removing any visible vegetal remain and shell, wet-sieved through a 2 mm sieve and treated by hydrogen peroxide (H_2_O_2_) 3% and distilled water 1:4 for 48 h. Grain size analysis (2000–0.2 μm) was performed through a Laser Diffraction Particle Size Analyzer (Malvern Mastersizer 2000) coupled with an autosampler. For further analyses we used only the clay percentage of the sample (particle size < 4 μm).

The average flooding depth in the saltmarshes considered was equal to 1.59 ± 1.03 cm (mean ± SD) while the clay content in the saltmarshes soil was on average equal to 19.55 ± 6.26% (Table 1).

### *Salicornia fruticosa* morphological and physiological traits

In each sampling plot, fourteen annual shoots of *S. fruticosa* were randomly collected from different individuals, sealed in plastic bags, stored in portable fridge, and transported to the laboratory, according to international protocol reference^79,80^. Five annual shoots were used to measure mean shoot length and fresh weight and later oven-dried at 70 °C for 72 h, weighed again (dry weight) and used for calculating the dry matter content of selected plant shoots.

The remaining nine annual shoots were used for quantifying chlorophyll, carotenoid, flavonoid and betacyanin content. The samples were pooled in three subsets (3 annual shoot × subset) and ground into fine powder under liquid nitrogen. Chlorophyll (i.e., chlorophyll *a* and *b*) and carotenoid concentration analysis was measured following Marchiol et al.^81^ protocol and expressed per gram fresh weight (μg g^−1^) (for the calculation methods see Wellburn^82^). Pheophytin content was estimated indirectly as a percentage compared to the amount of chlorophyll^83^. The flavonoid content was quantified according to the method described by Filippi et al*.*^84^ with minor changes and expressed as quercetin-eq content per gram fresh weight (μg quercetin-eq g^−1^).

The betacyanin concentration was determined by extraction from liquid nitrogen-pulverized samples using a 50 mM ascorbic acid in 80% methanol solution. The material was treated with a cold ultrasonic bath for 2 min, incubated for 30 min at − 20 °C and finally centrifuged at 15,000×*g* for 10 min at room temperature. A supernatant volume of 0.5 mL was diluted 1:1 with distilled water and its absorbance was measured at 538 nm. The determination of the betacyanin content was performed following Priatni & Pradita method ^85^.

### Plant community traits

The occurrence and the estimated cover value (% of plant cover compared to plot area) of all vascular plants were recorded in each plot. Species nomenclature followed Bartolucci et al*.*^86^. The identification of the plant material was performed by Francesco Boscutti, Paolo Cingano and Marco Vuerich in the field or in laboratory, using the national flora references^87^. All samplings were performed in accordance with national and international legislation^88,89^. None of the species employed in the study belong to any national and international protective species list nor to the IUCN Red List of endangered species. In particular, the amount of *S. fruticosa* samples collected was below the threshold limit set by regional legislation^89^. No voucher specimen of the plant material has been deposited in a publicly available herbarium. The height of 10 plants randomly selected of different species within each plot was measured in the field and subsequently the total aboveground plant biomass (all individuals of different species occurring) was harvested from a sub-plot (0.4 × 0.4 m), sealed in plastic bags, and transported in a portable fridge to the laboratory where it was fresh weighed, oven-dried at 70 °C for 72 h and weighed again (dry weight). Dry weight was then expressed as grams per square meters. Dry matter content was later calculated as the ratio of fresh weight to dry weight.

### UAV and remote sensing indices

The hexacopter Zephyr Exos UAV (Zephyr SRL, Forlì, Italy) equipped with a Parrot Sequoia multispectral camera (Parrot Drones SAS, Paris, France) was used to acquire multispectral images of each salt marshes. The hexacopter UAV was controlled via a hand-held remote controller, which sends waypoint navigation information to the aircraft allowing the hexacopter UAV to follow a flight path, at an altitude and speed defined by the user. The flight altitude above ground level was set at 40 m and the speed was 2 m s^−1^ for all nine flights. Each flight lasted for ~ 15 min, had an average total extent of 15.000 m^2^ and was performed close to solar noon (10.00 a.m./2.00 p.m.) with wind speed of < 1 m s^−1^, during the low-tide cycle concurrently to vegetation surveys and collection of plant and soil samples. Each flight path over the trial area was designed with an 85% forward and side overlap. The Parrot Sequoia multispectral camera has four sensors for four spectral bands: green (wavelength = 550 nm, bandwidth = 40 nm), red (wavelength = 660 nm, bandwidth = 40 nm), red-edge (wavelength = 735 nm, bandwidth = 10 nm), and near infrared (NIR) (wavelength = 790 nm, bandwidth = 40 nm). Before and after each flight was taken a picture of the calibrated reflectance panel (CRP), which is a Lambertian surface with a reflectance calibration curve associated that allows to convert raw pixels values into absolute reflectance. Moreover, to minimize the error during image capture due to change of the light, a downwelling light sensor (DLS) has been coupled to the multispectral camera to automatically adjust the readings to ambient light. For identifying each sampling site, two ground control points were located diagonally to the sampling site. Post-processing of the raw images was carried out using the Pix4Dmapper Pro software (version 4.0, PIX4D, Lausanne, Switzerland). Firstly, a geometric correction for each single-band raw image was performed using the camera calibration parameters included for each sensor. The post-processed images were then used to generate orthomosaic images including aligning photos, optimizing alignment, building a dense cloud, building digital surface models (DSMs) and building orthomosaics with a spatial resolution of 4 cm pixel^−1^. The orthomosaic images were radiometrically transformed to reflectance using the known reflectance of the CRP and with the DLS. Multispectral vegetation indices were calculated with the R package “terra”^90^. The calculated vegetation indices were: (i) Normalized Difference Vegetation Index (NDVI), (ii) Leaf Chlorophyll Index (LCI), (iii) Red Green Ratio Index (RGRI) and (iv) Anthocyanin Reflectance Index (ARI) (Table 4). The mean value of each vegetation index was calculated for each vegetation plot (2 × 2 m), as the average value of the pixel included in the plot.

**Table 4:** List, formula and brief interpretation description of the multispectral vegetation indices selected for the study.

Vegetation index and formula	Interpretation
$NDVI=\frac{NIR-RED}{NIR+RED}$	Estimation of geometrical features and green biomass production^94^
$LCI=\frac{NIR-RED EDGE}{NIR+RED}$	Estimation of chlorophyll content and distribution in leaves^95^
$RGRI= \frac{RED}{GREEN}$	Estimation of the course of leaf development in canopies and indicator of leaf production and stress^96,97^
$ARI = \frac{1}{GREEN}-\frac{1}{RED EDGE}$	Estimation of anthocyanin content in plant biomass^74^

### Statistical analysis

Prior to analysis, all pseudo replicates of the considered traits were averaged per each plot (n = 27). Plant community diversity was assessed by calculating the Shannon diversity index, based on the estimated cover value of the vascular plants recorded in each plot. The collinearity of the independent variables (*i.e.*, flooding depth, flooding duration, clay content) was tested by the Pearson correlation test. As flooding depth and flooding duration showed a significant positive correlation (r = 0.44, p = 0.02), we cautiously decided to test only for flooding depth effects. Clay content did not show any significant correlation with flooding (flooding depth vs soil clay: r = − 0.12, p = 0.54; flooding duration vs soil clay: r = − 0.25, p = 0.20). Linear mixed effects models (LMMs) were applied to examine the effect of flooding depth, clay content and their interaction (i.e., dependent variable ~ flooding depth × clay content) on community traits (i.e., plant height, dry weight, dry matter content, Shannon index), on *S. fruticosa* traits (i.e., shoot dry weight, shoot dry matter content, shoot length, chlorophyll, carotenoids, flavonoids and betacyanin content) and on vegetation indices (i.e., NDVI, LCI, RGRI, ARI). The salt marsh was considered as random factor. LMMs were applied using the “nlme” R package^91^. Model assumptions were verified using diagnostic plots and Shapiro–Wilk normality test (p > 0.05) on model residuals. Where model residuals violated any linear model assumption (i.e., shoot dry weight and betacyanin content), variables were log-transformed or evident outliers were discarded. The model interactions were manually removed when not significant (p > 0.05). Full model outcomes are reported in Table S1.

In order to assess the potential of remote sensing to upscale the community and species response, we assessed the contribution of each trait significantly varying along the studied gradients to the vegetation indices total variance. In particular, variance partitioning tools were used to quantify the vegetation indices (i.e., NDVI, LCI, RGRI, ARI) variance explained by community (i.e., plant height, dry weight) and *S. fruticosa* traits (i.e., shoot length, carotenoid and betacyanin content). At this stage we did not include any abiotic variable to better encompass the contribution of plant response to remote sending indices, in the upscaling perspective. Semi-partial coefficients of determination (part R^2^) to partition the variance explained by individual traits were calculated with R package “partR2” (version 0.9.1)^92^.

All graphs and statistical analyses were performed in R statistical software^93^.

### Supplementary Information

Supplementary Tables.

Supplementary Table S4.

## Data Availability

Data generated or analyzed during this study are included in this published article and its supplementary material file (Table S4).
