# Supplementary material for: New perspective for the upscaling of plant functional response to flooding stress in salt marshes using remote sensing
Source: Sci Rep. 2024 Mar 5;14:5472. doi: 10.1038/s41598-024-56165-4 (PMC10914724; doi:10.1038/s41598-024-56165-4)
Supplement: Supplementary file 1 — Supplementary Tables. [file 41598_2024_56165_MOESM1_ESM.docx]

**Supporting Information**

From plant traits to ecosystem: new perspective for the upscaling of salt marsh response to flooding stress with remote sensing tools

Marco Vuerich, Paolo Cingano, Giacomo Trotta, Elisa Petrussa, Enrico Braidot, Dora Scarpin, Annelore Bezzi, Michele Mestroni, Elisa Pellegrini and Francesco Boscutti

**Table S1.** Results of the LMMs relating the salt marsh community traits (i.e., plant height, total DW, total DMC, Shannon index), the *S. fruticosa* traits (i.e., shoot DW, shoot DMC, shoot length, chlorophyll, carotenoids, flavonoids, betacyanin content) the vegetation indices (i.e., NDVI, LCI, RGRI, ARI) with flooding depth, soil clay content and their interaction. Significant relationships are in bold. Degrees of freedom (Df), Fisher value (F-value) and p-values are shown.

| **Dependent variable** | **Independent variable** | **Df** | **Estimate** | **SE** | **t-Value** | **p-Value** |
| --- | --- | --- | --- | --- | --- | --- |
| *Plant height* | Flooding depth | 1,15 | 0.46 | 5.2 | 0.08 | 0.931 |
|  | Clay content | 1,15 | 0.38 | 0.3 | 1.32 | 0.205 |
|  | Flooding depth*Clay content | 1,15 | -0.24 | 0.3 | -0.85 | 0.405 |
| *Total DW* | Flooding depth | 1,15 | -39.78 | 42.3 | -0.94 | 0.361 |
|  | Clay content | 1,15 | 1.12 | 2.5 | 0.44 | 0.664 |
|  | Flooding depth*Clay content | 1,15 | 0.81 | 2.1 | 0.39 | 0.705 |
| *Total DMC* | Flooding depth | 1,15 | -13.98 | 51.54 | -0.27 | 0.790 |
|  | Clay content | 1,15 | 1.23 | 3.09 | 0.39 | 0.696 |
|  | Flooding depth*Clay content | 1,15 | 0.87 | 2.59 | 0.34 | 0.741 |
| *Shannon index* | Flooding depth | 1,15 | -0.11 | 0.29 | -0.38 | 0.706 |
|  | Clay content | 1,15 | -0.01 | 0.01 | -0.58 | 0.567 |
|  | Flooding depth*Clay content | 1,15 | -0.00 | 0.01 | -0.00 | 0.998 |
| *log(Shoot DW)* | Flooding depth | 1,15 | -0.04 | 0.25 | -0.16 | 0.875 |
|  | Clay content | 1,15 | 0.01 | 0.01 | 0.90 | 0.383 |
|  | Flooding depth*Clay content | 1,15 | -0.00 | 0.01 | -0.30 | 0.769 |
| *Shoot DMC* | Flooding depth | 1,15 | -5.02 | 13.98 | -0.36 | 0.724 |
|  | Clay content | 1,15 | -0.07 | 0.78 | -0.09 | 0.929 |
|  | Flooding depth*Clay content | 1,15 | 0.21 | 0.74 | 0.28 | 0.780 |
| *Shoot length* | Flooding depth | 1,14 | 1.18 | 0.54 | 2.20 | **0.046** |
|  | Clay content | 1,14 | 0.15 | 0.03 | 4.69 | **< 0.001** |
|  | Flooding depth*Clay content | 1,14 | -0.08 | 0.03 | -2.77 | **0.015** |
| *Chlorophyll* | Flooding depth | 1,15 | 128.12 | 97.21 | 1.32 | 0.207 |
|  | Clay content | 1,15 | 1.69 | 5.31 | 0.32 | 0.755 |
|  | Flooding depth*Clay content | 1,15 | -7.24 | 5.24 | -1.38 | 0.187 |
| *Carotenoids* | Flooding depth | 1,12 | -31.19 | 8.93 | -3.49 | **0.004** |
|  | Clay content | 1,12 | -0.85 | 0.44 | -1.94 | **0.076** |
|  | Flooding depth*Clay content | 1,12 | 1.56 | 0.48 | 3.29 | **0.007** |
| *Flavonoids* | Flooding depth | 1,15 | -5.21 | 7.27 | -0.71 | 0.484 |
|  | Clay content | 1,15 | -0.57 | 0.43 | -1.33 | 0.203 |
|  | Flooding depth*Clay content | 1,15 | 0.20 | 0.37 | 0.552 | 0.589 |
| *log(Betacyanin)* | Flooding depth | 1,15 | 0.47 | 0.72 | 0.81 | 0.430 |
|  | Clay content | 1,15 | 0.05 | 0.03 | 1.46 | **0.164** |
|  | Flooding depth*Clay content | 1,15 | -0.01 | 0.03 | -0.41 | 0.684 |
| *NDVI* | Flooding depth | 1,15 | 0.05 | 0.05 | 1.05 | 0.310 |
|  | Clay content | 1,15 | 0.00 | 0.00 | 1.53 | 0.147 |
|  | Flooding depth*Clay content | 1,15 | -0.00 | 0.00 | -1.89 | 0.077 |
| *LCI* | Flooding depth | 1,15 | 0.04 | 0.03 | 1.48 | 0.159 |
|  | Clay content | 1,15 | 0.00 | 0.00 | 1.75 | 0.100 |
|  | Flooding depth*Clay content | 1,15 | -0.00 | 0.00 | -2.23 | **0.041** |
| *RGRI* | Flooding depth | 1,15 | 0.01 | 0.05 | 0.16 | 0.879 |
|  | Clay content | 1,15 | 0.00 | 0.00 | 0.19 | 0.852 |
|  | Flooding depth*Clay content | 1,15 | 0.00 | 0.00 | 0.69 | 0.495 |
| *ARI* | Flooding depth | 1,15 | 1.73 | 0.89 | 1.94 | 0.071 |
|  | Clay content | 1,15 | 0.04 | 0.05 | 0.87 | 0.396 |
|  | Flooding depth*Clay content | 1,15 | -0.11 | 0.05 | -2.37 | **0.031** |

**Table S2.** Results of the LMMs relating the vegetation indices (i.e., NDVI, LCI, RGRI, ARI) with flooding depth, soil clay content and their interaction. Significant relationships are in bold. Degrees of freedom (Df), Fisher value (F-value) and p-values are shown.

| **Dependent variable** | | **Independent variable** | | | | **Df** | | | **Estimate** | | | **SE** | | | **t-Value** | | | **p-Value** | | |
| --- | --- | --- | --- | --- | --- | --- | --- | --- | --- | --- | --- | --- | --- | --- | --- | --- | --- | --- | --- | --- |
| *NDVI* | | | | Flooding | | 1,16 | | | -0.04 | | | 0.01 | | | -3.14 | | | **0.006** | | |
|  | | | | Clay content | | 1,16 | | | 0.00 | | | 0.00 | | | 0.17 | | | 0.865 | | |
| *LCI* | | | | Flooding depth | | 1,15 | | | 0.04 | | | 0.03 | | | 1.48 | | | 0.159 | | |
|  | | | | Clay content | | 1,15 | | | 0.00 | | | 0.00 | | | 1.75 | | | 0.100 | | |
|  | | | | Flooding depth*Clay content | | 1,15 | | | -0.00 | | | 0.00 | | | -2.23 | | | **0.041** | | |
| *RGRI* | | | Flooding | | | 1,16 | | | 0.04 | | | 0.01 | | | 3.49 | | | **0.003** | | |
|  | | | Clay content | | | 1,16 | | | 0.00 | | | 0.00 | | | 1.06 | | | 0.303 | | |
| *ARI* | | | Flooding depth | | | 1,15 | | | 1.73 | | | 0.89 | | | 1.94 | | | 0.071 | | |
|  | | | Clay content | | | 1,15 | | | 0.04 | | | 0.05 | | | 0.87 | | | 0.396 | | |
|  | | | Flooding depth*Clay content | | | 1,15 | | | -0.11 | | | 0.05 | | | -2.37 | | | **0.031** | | |

**Table S3.** Contribution of salt marsh plant community traits and S. fruticosa traits to vegetation indices. Proportions of vegetation indices (i.e., NDVI, LCI, RGRI, ARI) variance explained by plant community traits (i.e., plant height and total DW) and S. fruticosa traits (i.e., shoot length, carotenoid and betacyanin content).

| **Dependent variable** | **Independent variables** | **Df** | **t Value** | ***p*-Value** | **Part-R^2^** |
| --- | --- | --- | --- | --- | --- |
| NDVI | Plant height | 1,11 | 0.78 | 0.448 | 0.00 |
|  | Total DW | 1,11 | -0.76 | 0.456 | 0.00 |
|  | Shoot length | 1,11 | 2.83 | 0.014 | 0.10 |
|  | Carotenoid | 1,11 | 1.30 | 0.212 | 0.01 |
|  | Betacyanin | 1,11 | -2.33 | 0.036 | 0.08 |
| LCI | Plant height | 1,12 | 0.22 | 0.828 | 0.00 |
|  | Total DW | 1,12 | -1.86 | 0.085 | 0.01 |
|  | Shoot length | 1,12 | 2.94 | 0.011 | 0.12 |
|  | Carotenoid | 1,12 | 2.04 | 0.062 | 0.06 |
|  | Betacyanin | 1,12 | -1.81 | 0.093 | 0.05 |
| RGRI | Plant height | 1,11 | 0.54 | 0.592 | 0.03 |
|  | Total DW | 1,11 | -0.47 | 0.644 | 0.02 |
|  | Shoot length | 1,11 | -1.99 | 0.068 | 0.12 |
|  | Carotenoid | 1,11 | -1.91 | 0.079 | 0.10 |
|  | Betacyanin | 1,11 | 1.57 | 0.140 | 0.10 |
| ARI | Plant height | 1,11 | 0.82 | 0.424 | 0.03 |
|  | Total DW | 1,11 | -2.34 | 0.036 | 0.06 |
|  | Shoot length | 1,11 | 2.24 | 0.043 | 0.00 |
|  | Carotenoid | 1,11 | -1.61 | 0.162 | 0.06 |
|  | Betacyanin | 1,11 | -0.75 | 0.467 | 0.00 |

**Table S4.** Dataset containing all data generated and analyzed during the study (table included as Excel file in supplementary material).
